# Case Report: Transient Increase of CMR T1 Mapping Indices in a Patient With COVID-19 mRNA Vaccine Induced Acute Myocarditis

**DOI:** 10.3389/fcvm.2022.880717

**Published:** 2022-04-27

**Authors:** Uzair Ansari, Simone Britsch, Sebastian Rogowski, Daniel Duerschmied, Theano Papavassiliu

**Affiliations:** ^1^First Department of Medicine, Faculty of Medicine Mannheim, University Medical Center Mannheim, University of Heidelberg, Mannheim, Germany; ^2^European Center for AngioScience, Mannheim, Germany; ^3^DZHK (German Center for Cardiovascular Research) Partner Site Heidelberg/Mannheim, Mannheim, Germany; ^4^Klinik München Bogenhausen, München, Germany

**Keywords:** cardiac magnetic resonance imaging, myocarditis, COVID-19, T1 mapping, mRNA vaccine

## Abstract

**Background:**

Acute myocarditis is commonly associated with viral infections, including severe acute respiratory syndrome coronavirus-2 (SARS-CoV-2). Myocarditis following mRNA COVID-19 vaccination has also been reported, however this is rare and usually resolves within days or weeks. We present a case of acute myocarditis reported after vaccination with mRNA-1273 COVID-19 vaccine (Moderna) diagnosed using cardiac magnetic resonance imaging (CMR). This report describes the utility of CMR in the diagnosis and follow-up of such patients using parameters which could suggest the clinical course of myocarditis.

**Case Summary:**

A 23-year-old male presented in the emergency department with complaints of chest pain radiating to the left arm following vaccination with the second dose of COVID-19 mRNA-1273 vaccine (Moderna). Patient's history revealed an incidence of myocarditis in the past. CMR showed a mid-range left ventricular ejection fraction (38%) and subepicardial late gadolinium enhancement (LGE) in the inferolateral and apical myocardial segments with diffuse elevation of native T1 mapping relaxation times in all myocardial segments. The patient was admitted briefly in the intensive care unit and after a favorable clinical course was discharged from the hospital in stable condition. A follow-up CMR after 3 months revealed normalization of LVEF (57%) and native T1- times in most segments. Scarred myocardium reflecting chronic myocarditis continued to show elevated T1 times.

**Conclusions:**

Our patient presenting with acute myocarditis after recent COVID-19 mRNA vaccination reported a favorable clinical course. CMR revealed increased T1 mapping relaxation times diffusely spread across the myocardium and an impairment of the left ventricular function (LVEF) during the acute phase. However, the LVEF as well as the T1 times normalized at follow-up in all segments except for myocardium affected by chronic myocarditis.

## Introduction

The use of BNT163b2 mRNA (Pfizer-Biontech) and mRNA-1273 (Moderna) vaccines to curb the spread of severe acute respiratory syndrome coronavirus 2 (SARS-CoV-2) infection has gained traction worldwide (1). These mRNA vaccines have proven to be remarkably effective in preventing symptomatic infection and severe illness associated with coronavirus disease 2019 (COVID-19). However, isolated reports have suggested their use is associated with an increased incidence of myocarditis and pericarditis (2, 3). The role of cardiovascular magnetic resonance imaging (CMR) in the diagnosis of acute myocarditis has already been established (4). We present a patient with acute myocarditis following vaccination with mRNA-1273 (Moderna), thereby defining the role of CMR at index presentation and follow-up in such patients.

## Case Presentation

A 23-year-old male initially presented in our emergency department with symptoms suggestive of angina pectoris. The patient reported the sudden onset of chest pain radiating to the left arm as well as headache 1 day after vaccination with the second dose of the mRNA-1273 (Moderna) COVID-19 vaccine. Dyspnoea, fever, or excessive sweating were denied. Further examination revealed a relevant past-history of perimyocarditis in 2018 and 2019 (possibly post-infectious). The patient was not on medication at the time of presentation.

The clinical examination of the patient was unremarkable. The body temperature recording was 37.5°C. The heart rate on admission was 96 beats/min and the blood pressure was 110/60 mmHg. The oxygen saturation was 99% on room air. An electrocardiogram (ECG) showed sinus rhythm with mild concave ST-elevations in II, III, and aVF. Laboratory data (see Table 1) revealed leucocytosis, elevated levels of creatine kinase, C-reactive protein, and high-sensitivity troponin I levels. The nasopharyngeal SARS-CoV-2 PCR test was negative, and the patient denied any history of infection with COVID-19. The patient was admitted to our intensive care unit (ICU) for observation and further clinical management.

**Table 1:** Key laboratory findings supporting the diagnosis.

**Laboratory test**	**Peak values**	**Reference range**
WBC (10^9^/L)	13.60	4.2–10.2
Hb (g/dL)	14.3	13.2–16.7
Creatine kinase (U/L)	416	46–171
hs-troponin I (μg/L)	10.923	0–0.045
NT-proBNP (ng/L)	1970	0–125
Lactate dehydrogenase (U/L)	281	120–246
C-reactive protein (mg/L)	77	0–5

The troponin I level peaked on the second day (10.923 μg/L; normal range 0–0.045 μg/L) and NT-proBNP levels showed moderate elevation (1,970 ng/L; normal range 0–125 ng/L). A thoracic CT revealed no obvious pulmonary infiltrates and no evidence of coronary plaques or significant stenoses in the coronaries. An echocardiogram performed in the ICU revealed a moderately reduced left ventricular ejection fraction (LVEF) with hypokinetic inferolateral and apical segments.

**Figure 1:**
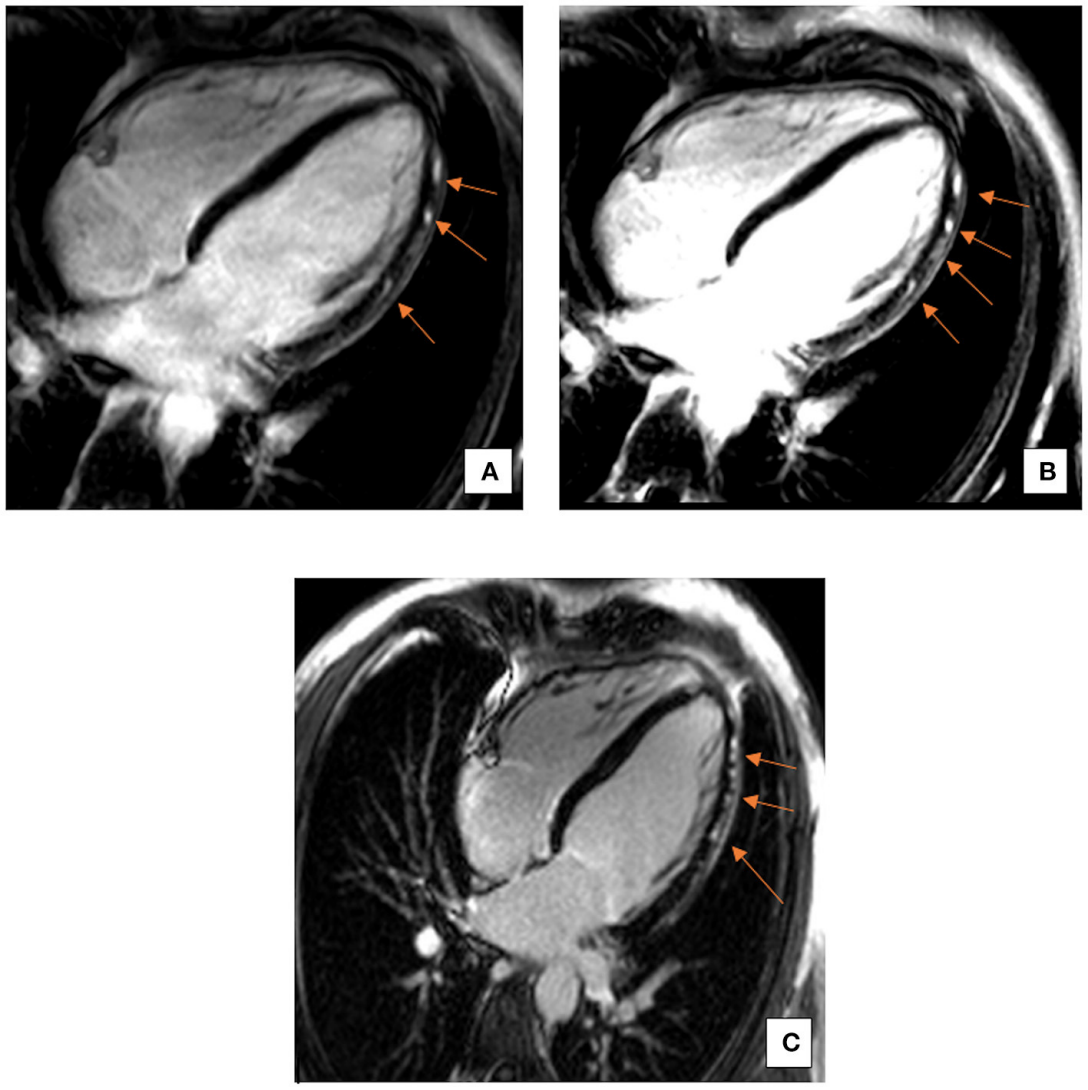
Representative 4-Chamber (4CH) view images of late gadolinium enhancement (LGE). A comparison between LGE images from the past bout of myocarditis **(A,B)** and the current acute phase **(C)**. LGE is comparable and restricted to the subepicardial regions, particularly in the inferolateral and apical myocardial segments. Arrows indicate areas of LGE.

**Figure 2:**
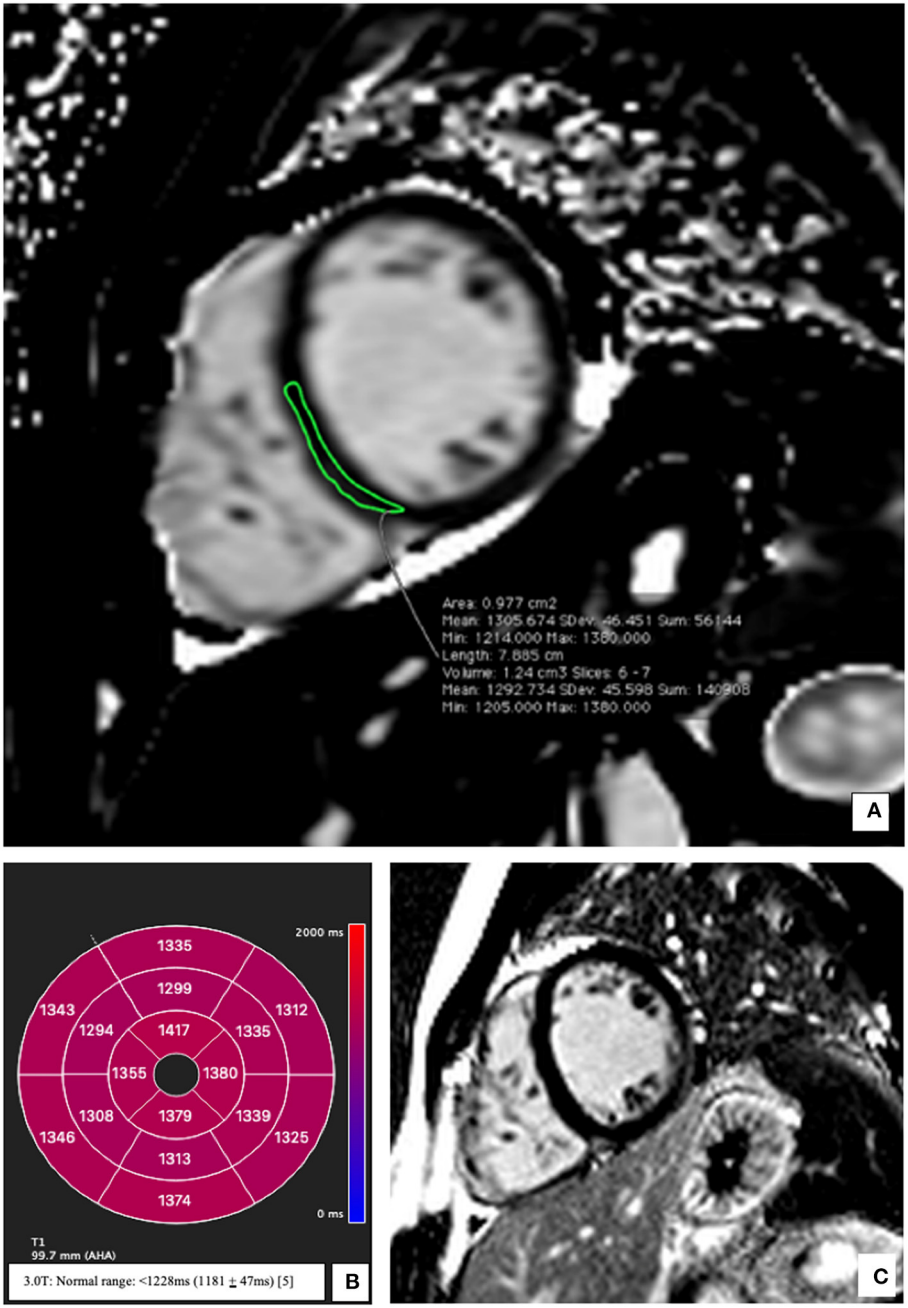
Representative native T1 mapping image and the corresponding late gadolinium enhancement (LGE) image at presentation. **(A)** Midventricular short axis image of T1 map (SAX) showing elevation of the T1 times; an example from the inferoseptal segment (demarcated green area: 1,305 ms). **(B)** Bullseye plot showing the native T1-times. Segmentation was performed according to the AHA 16-segment model. Native T1 times were increased in all segments irrespective of the presence/absence of LGE. **(C)** Corresponding mid-ventricular SAX LGE image without evidence of LGE. Normal T1 Mapping values for a 3 Tesla MRI <1,228 ms (1,181 ± 47 ms) (5).

The echocardiographic findings were confirmed using a cardiac MRI (CMR) (3T MAGENTOM SKYRA, Siemens Healthineers, Erlangen, Germany). Considering the medical history of the patient, images from the CMR scan during the earlier bout of myocarditis (Figures 1A,B) were compared to the present (Figure 1C). The contrast enhanced images showed comparable subepicardial late gadolinium enhancement (LGE) in the lateral and apical myocardial wall during the qualitative assessment. A definitive diagnostic conclusion based on LGE alone could not be drawn due to inter-scanner and inter-study differences. Cine images from the current CMR revealed a dilated left ventricle (end-diastolic diameter-−64 mm) and a moderately reduced LVEF (38%) vs. a mildly reduced LVEF (51%) in the examination 2 years ago. Additionally, in the current CMR, native T1 maps revealed a diffuse increase in relaxation times in all myocardial segments [1,344 ± 74 ms; normal range <1,228 ms (1,181 ± 47 ms) for this 3T machine] (5). As example, the elevation of T1 mapping indices in the mid-ventricular myocardial inferoseptal segment has been shown in Figure 2, although there is no evidence of any LGE in this segment. There was evidence of a mild pericardial effusion (3 mm). This could suggest renewed involvement of affected myocardium with spread of acute inflammation in other segments too. These findings support the diagnosis of acute myocarditis according to the updated Lake Louise criteria (4).

**Figure 3:**
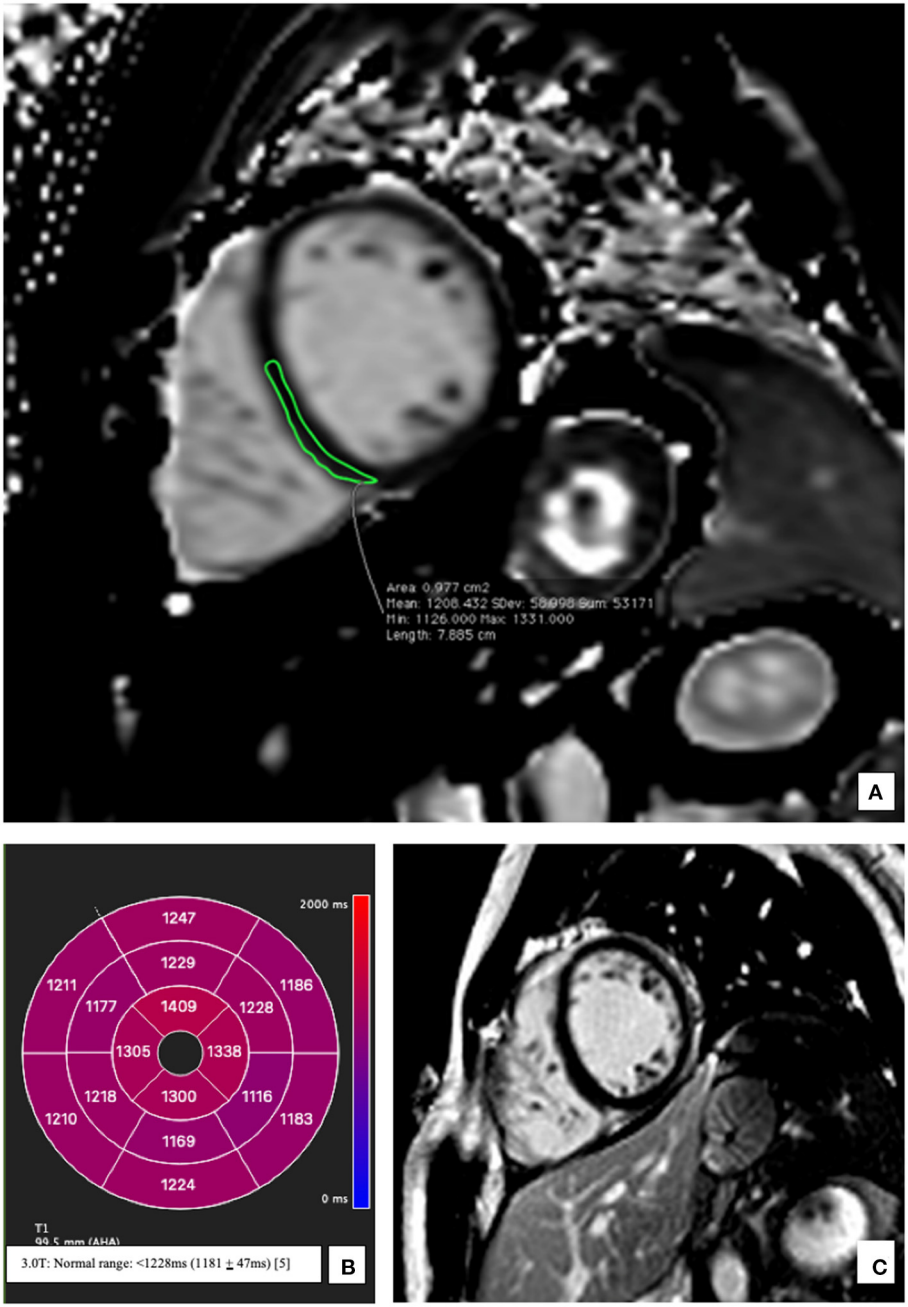
Representative native T1 mapping image and the corresponding late gadolinium enhancement (LGE) image at follow up after 3 months. **(A)** Midventricular short axis image (SAX) of T1 map showing normal T1 times at follow up; an example from the inferoseptal segment (demarcated green area: 1,208 ms) **(B)** Bullseye plot showing the native T1-times. Segmentation was performed according to the AHA 16-segment model. Native T1 times show a return to baseline at follow-up except for values in apical myocardium which correspond to the residual scar tissue reflecting chronic myocarditis **(C)** Corresponding mid-ventricular SAX LGE image still without evidence of LGE.

The patient was started on a therapy with Ibuprofen 400 mg (twice daily), beta-blockers (Bisoprolol 2.5 mg once daily) as well as an ACE-Inhibitor (Ramipril 2.5 mg once daily). There was rapid improvement of clinical symptoms and a repeat echocardiogram performed on day 6 showed only a mildly reduced LVEF (52%) thus facilitating a timely discharge. The patient was stable throughout the course of hospital stay and no complications were documented. A follow-up CMR performed after 3 months revealed a markedly improved LVEF (57%). Videos documenting this improvement have been added as Supplementary Material. LGE was comparable to the previous studies. T1 mapping indices had normalized (1,194 ms) except for myocardial segments corelating to chronic myocarditis (also evident in past CMR images) (Figure 3).

## Discussion

The occurrence of post-vaccination myocarditis has most notably been associated with the smallpox vaccine; however, isolated cases have also been reported with the use of influenza, tetanus, human papillomavirus, and hepatitis B vaccines (6). Although the pathophysiological process describing this entity is still not clearly understood, limited endomyocardial biopsy studies have suggested lymphocytic and eosinophilic infiltration adjacent to necrotic myositis suggestive of an immune-mediated injury (7). In patients reporting myocarditis after vaccination with an mRNA vaccine, it is hypothesized that a non-specific innate inflammatory response or a molecular mimicry mechanism between viral spike protein and an unknown cardiac protein leads to cardiac injury (1). Myocarditis following SARS-CoV-2 mRNA vaccination has increasingly been observed in young male adults within 1 week of viral antigen-induced immune activation. The increased propensity of myocarditis in young adults following the second dose of vaccine lends further support to the hypothesis suggesting a vaccine-associated maladaptive immune response (8).

A recent study suggested that the onset of clinical myocarditis symptoms (after exposure to an immunological trigger) was probably shorter for COVID-19 vaccine associated cases as compared to those reported after a viral illness. Presenting symptoms also appeared to resolve earlier in the post-vaccination myocarditis group (9). These results could reflect a self-limiting pattern of cardiac injury confined to a small subgroup of post-vaccinated young adults, as also seen in our patient.

Different regimens have been employed to treat post-vaccination myocarditis. Our patient received a non-steroidal anti-inflammatory drug (NSAID) in addition to a beta-blocker and an ACE-Inhibitor. This led to the successful resolution of clinical symptoms and complete recovery on follow-up. In one case series, patients were administered colchicine or prednisolone, which also showed similar favorable outcomes (1).

CMR has proven to be an essential tool in the clinical management of myocarditis. In a recent multicenter study, it could be shown that myocardial injury during acute COVID-19 infection requiring hospital admission was associated with a CMR abnormality in approximately half of the patients. Additionally, almost 27% had pathological CMR findings consistent with a non-infarct myocarditis pattern of injury (10). The use of CMR in the current scenario is especially advantageous. Case reports have suggested that CMR studies in patients with post-COVID-19 vaccine myocarditis could reveal resolution of edema, with possible evidence of residual myocardial scarring (11, 12). Furthermore, the diagnosis of post-vaccination myocarditis reflected by a transient rise in T1 mapping indices in our patient also has prognostic relevance.

A study by Ferreira et al., has suggested that the diffuse increase of T1 mapping indices is influenced by myocardial edema seen in acute myocarditis. Additionally, the acute inflammation as revealed by T1 mapping was shown to be superior to edema imaging with T2-weighted sequences (13). An increase in T1 mapping indices could also be transitory, helping differentiate between acute and chronic myocardial inflammation (14). Our patient also showed a transient increase in T1 mapping indices. Most segmental values normalized at follow-up, however, residual inflammation (reflected by increased T1 mapping values) continued to be seen in the apex, which could be suggestive of chronic myocarditis. The continued presence of LGE in these inferolateral and apical segments also strengthens this observation.

The Vaccine Adverse Event Reporting System (VAERS) has already received 1226 preliminary reports of myocarditis and pericarditis after about 300 million doses of the Pfizer and Moderna vaccines (until mid-2021) (15). A recent study highlighting the adverse events associated with the use of mRNA COVID-19 vaccines (BNT162b2 mRNA) outlined the risk of acute kidney injury, arrhythmias, deep vein thrombosis, pulmonary embolism and Bell's palsy in addition to myocarditis. Interestingly, the risk of myocarditis after vaccination was reported to increase by a factor of 3, which translated to 3 excess events per 100,000 persons. This risk difference per 100,000 among those infected with SARS-CoV-2 was reported to be 11 (16). These results emphasize the favorable benefit-risk assessment for COVID-19 mRNA vaccination in all adult age groups (17).

Recently, the increased incidence of myocarditis after vaccination with mRNA-1273 COVID-19 vaccine (Moderna) had led many countries in western Europe to temporarily suspend its use in adults under 30 years of age. This case study highlights the clinical course of one such patient diagnosed with post-vaccination myocarditis (Moderna). The transient increase in native T1 mapping, as well as the transient LVEF deterioration during the acute phase reflects a similar pattern of a self-limiting and reversible cardiac injury. This underscores the favorable benefit-risk assessment for COVID-19 mRNA vaccination and the importance of CMR in the clinical management of these patients.

## Conclusion

This case report reaffirms the important role of CMR in the diagnosis of post-vaccination myocarditis. There was a transient increase in native T1 mapping relaxation times, as well as a transient LVEF deterioration during the acute phase of myocarditis in our patient, which was induced by inoculation with the COVID-19 mRNA-1273 vaccine (Moderna). Nevertheless, these values normalized at follow-up in all segments except for residual myocardium affected by chronic myocarditis.

## Limitations

This case report is not reflective of an entire study population. Large multi-centric trials would be required to establish the true nature of adverse effects of COVID-19 mRNA vaccines. Furthermore, the follow-up period would need to be considerably longer to draw any definitive conclusions.

## Data Availability Statement

The raw data supporting the conclusions of this article will be made available by the authors, without undue reservation.

## Ethics Statement

The studies involving human participants were reviewed and approved by Medizinische Ethikkommmision II, Medizinische Fakultät Mannheim, Universität Heidelberg. The patients/participants provided their written informed consent to participate in this study. Written informed consent was obtained from the individual(s) for the publication of any potentially identifiable images or data included in this article.

## Disclosure

The authors take responsibility for all aspects of the reliability and freedom from bias of the data presented and their discussed interpretation.

## Author Contributions

UA and TP wrote the article. UA, TP, SR, SB, and DD analyzed the data, discussed the results, and reviewed and approved the article. All authors contributed to the report and approved the submitted version.

## Conflict of Interest

The authors declare that the research was conducted in the absence of any commercial or financial relationships that could be construed as a potential conflict of interest.

## Publisher's Note

All claims expressed in this article are solely those of the authors and do not necessarily represent those of their affiliated organizations, or those of the publisher, the editors and the reviewers. Any product that may be evaluated in this article, or claim that may be made by its manufacturer, is not guaranteed or endorsed by the publisher.
